# Water Stress and Shade Elicit Similar Convergent Responses in Maize Kernel Development and Gene Expression During Early Post-Pollination

**DOI:** 10.3390/plants15142130

**Published:** 2026-07-10

**Authors:** Tim L. Setter, Annemiek Morrison

**Affiliations:** 1 Section of Soil and Crop Sciences, School of Integrative Plant Science, Cornell University, Ithaca, NY 14853, USA; 2 Agricultural Sciences Program, Cornell University, Ithaca, NY 14853, USA; acm285@cornell.edu

**Keywords:** kernel set, kernel abortion, transcript profiling, photosynthate partitioning, carbohydrate metabolism, trehalose, abscisic acid, ethylene, pedicel-placenta, endosperm

## Abstract

Maize crop yields are substantially diminished by drought during early phases of kernel development. Kernel development is also affected by stresses which limit photosynthate availability, such as high plant density and shade. To advance our understanding of the mechanisms by which these stresses affect kernel development, we subjected potted maize plants in the greenhouse to water stress and shade from 1 to 9 days after pollination and determined evapotranspiration, root growth in deep pots, carbohydrates and ABA in leaves and kernel tissues, and profiled expression of gene transcripts in kernel tissues with RNA-seq. Root development was decreased by shade, whereas it was increased by severe water stress (WS). Both severe WS and shade increased ABA levels in kernel tissues. A large fraction of differentially expressed genes in pedicel-placenta tissues of kernels were affected similarly by severe WS and shade, including genes involved in synthesis and response to ABA, carbohydrates, trehalose, and ethylene. Differential expression in response to mild WS was intermediate or minimal. In endosperm-nucellus, expression of genes in these categories was strongly affected by shade, but less so by severe WS. We conclude that the similarity of ABA accumulation and gene expression in pedicel-placenta of severe WS and shade indicates that this tissue likely plays a pivotal role in kernel set decisions.

## 1. Introduction

In maize (*Zea mays* L.), grain yield is vulnerable to drought at flowering and during the initial period after pollination [1,2,3]. Drought stress at this phase of development primarily affects yield by decreasing the fraction of florets that develop fully mature kernels (kernel set). Nevertheless, maize yield in drought environments has been substantially improved by targeting this vulnerable phase and breeding for improved kernel development and yield in response to managed stress imposed at flowering [4,5,6,7,8,9]. An improved understanding of the mechanisms by which kernel tissues respond to water stress (WS) and diminished photosynthate supply would assist efforts to further improve crop performance via genetic or management approaches.

While research into water deficit has often relied on controlled greenhouse environments, where results may not always translate directly to field performance, a substantial body of work has been conducted under agronomic field conditions. These field studies have confirmed that the magnitude of kernel set loss is positively correlated with declines in photosynthesis [10] and the influx of photosynthate into kernels [11,12,13,14]. Although floral and kernel tissues represent a small sink for photosynthate during pre-pollination and the first few days of post-pollination growth, kernel set responds to current photosynthate availability. In addition to water deficit, a range of other treatments that decrease photosynthesis also decrease kernel set, including canopy shade [12,13,15,16], high population density [17], nitrogen deficiency [18,19,20], leaf diseases [21], and defoliation [22]. Although maize kernel set is diminished by water deficit imposed at flowering, it is substantially ameliorated by feeding exogenous photosynthate (sucrose solution) via stem infusion [23,24,25].

While numerous studies support the hypothesis that photosynthate may serve as a signal for decisions on growth arrest vs abortion, other signals may also play a role. Several studies have shown that water deficit increases ABA concentration in reproductive tissues in which kernel set is diminished and that exogenous application of ABA decreases kernel growth and cell division in endosperms in their first few days after pollination [26,27,28,29,30]. Others have suggested that loss of expansive growth during WS rather than a change in carbon status is the initiating factor that leads to growth arrest and kernel abortion [31,32]. Expansion growth, which depends on development of cell turgor to drive cell wall plastic deformation and expansion, is diminished when tissue water potential is decreased during WS. ABA synthesis is also well known to be induced when tissue water potential declines. However, these changes are not known to occur in response to other stresses which affect photosynthate status, such as shade. Conversely, shade, and stresses that create severe carbohydrate deprivation, such as hypoxia, also induce ethylene synthesis which can also interact with other signaling systems to result in multifaceted metabolic and tissue developmental responses [33,34], including loss of kernel set in maize [12,35,36].

Our objective was to perform a side-by-side comparison of WS and shade treatments at early post-pollination to determine which mechanistic factors distinguish or are in common between these stresses. Given the potential that important signaling would occur in the phloem unloading and sugar flux pathway, as well as in growing tissues, we used RNA-sequencing to profile gene expression in pedicel-placenta and endosperm-nucellus tissues.

## 2. Results

### 2.1. Evapotranspiration, Leaf ABA and Leaf Carbohydrate Responses to Stress

**Figure 1:**
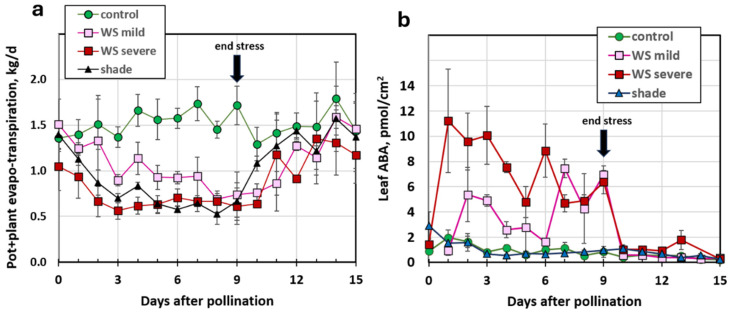
Evapotranspiration rate (**a**) and leaf ABA content per cm^2^ of leaf area (**b**) in plants subjected to control, WS (water stress) mild, WS severe, and shade treatments from 0 to 9 days after pollination (DAP). Averages ± SEM are shown.

Water stress and shade treatments were imposed from the day of pollination to 9 days after pollination (0–9 DAP) so that treatments coincided with the time of initial endosperm cell division and kernel development. In the severe water stress treatment, irrigation was withheld starting at one day before pollination, whereas in the mild water stress irrigation was withheld starting one day after pollination. Evapotranspiration in water stressed plants declined gradually during the first 3–4 days as the plants depleted soil water and stomatal closure restricted water loss (Figure 1a). Evapotranspiration also declined in the shade treatment as radiation limited energy for evapotranspiration, and stomata closed in response to low light flux density. From 3 to 9 DAP, the well-watered controls evapotranspired 1.58 ± 0.10 kg H_2_O/d, whereas the shade and severe WS treatments used 0.65 ± 0.03 and 0.64 ± 0.09 kg/d, respectively. The mild WS used 0.89 ± 0.04 kg H_2_O/d. After ending the WS and shade treatments at 9 DAP, the rate of water use in stressed plants gradually increased such that at 12–15 DAP they were not significantly different from controls. Leaf ABA levels reflected the severity of stress (Figure 1b). From 1 to 9 DAP, severe WS leaves averaged 7.8 ± 1.1 pmol ABA/cm^2^ while mild WS leaves averaged 4.5 ± 0.6 pmol/cm^2^. Leaves in the well-watered control and shade treatments averaged 1.1 ± 0.2 and 0.94 ± 0.12 pmol/cm^2^, respectively.

**Figure 2:**
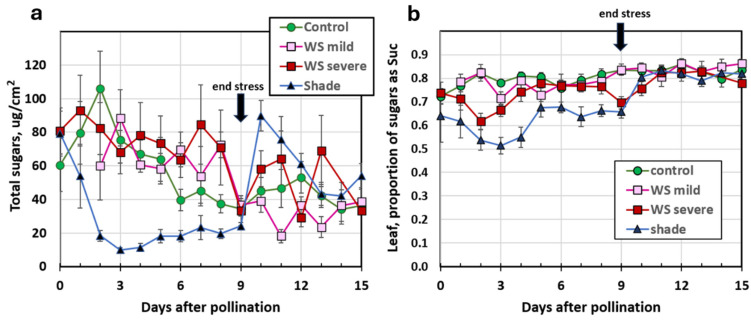
Total leaf sugar content per cm^2^ leaf area (**a**) and the proportion of leaf sugars as sucrose (**b**) in plants subjected to control, WS mild, WS severe, and shade treatments from 0 to 9 DAP. Averages ± SEM are shown.

Leaf sugar content was quite variable during the stress and recovery phases in all treatments except shade (Figure 2a). Such variability was likely due to day-to-day differences in sunlight in the greenhouse and other unidentified factors. In the shade treatment, leaf sugar levels declined considerably during stress, then returned to control levels after plants were returned to full light (Figure 2b). Shaded plants also differed from other treatments in having a lower proportion of sugar as sucrose during the shade stress, then returning to control levels after the stress was relieved.

### 2.2. Root Growth Responses to Stress

**Figure 3:**
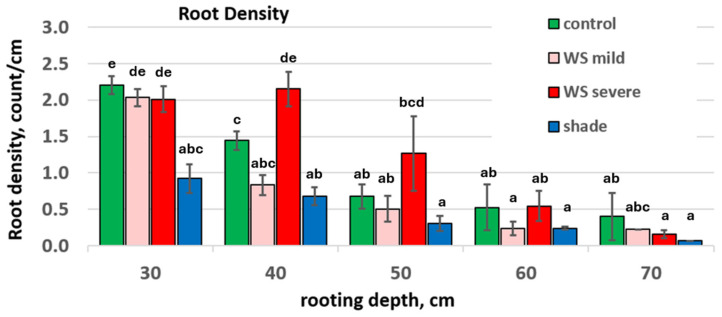
Root density per cm of soil transects at various depths of soil to which pots were transplanted at one day before pollination. Plants were subjected to control, WS mild, WS severe, and shade treatments from 0 to 9 DAP; density was assessed via pot transparent windows at 9 DAP. Averages ± SEM are shown; comparisons between treatments at various depths that do not have the same letter are significantly (*p* ≤ 0.05) different using the Tukey HSD multiple range test.

We designed the experiment to simulate conditions in the field where water depletion in upper soil layers is followed by root development and deeper rooting to access additional water. On the day of pollination, we transplanted each plant and its undisturbed root mass onto a deep (1 m) bed of moist soil which was precisely wetted to provide a limited quantity of water (1.8 kg) with an initial soil water potential of −0.18 MPa. At 9 DAP, at least a few roots in all treatments had grown the full 1 m to the bottom of the deep pots. However, roots in the severe WS treatment had a significantly (*p* ≤ 0.05) higher root density compared to the control and other stress treatments at the 40 cm depth and appeared to exceed other treatments at 50 cm (Figure 3). In contrast, root density in the shade treatment was significantly (*p* ≤ 0.05) lower than the other treatments at 30 cm and tended to be the lowest at all depths. These results are consistent with the expectation that a limited amount of photosynthate in the shade treatment limited root growth and that, despite a lower rate of photosynthesis in the severe water stress, partitioning of limited photosynthate favored root growth.

### 2.3. Stress Effects on Kernel Number and Size at Maturity

**Figure 4:**
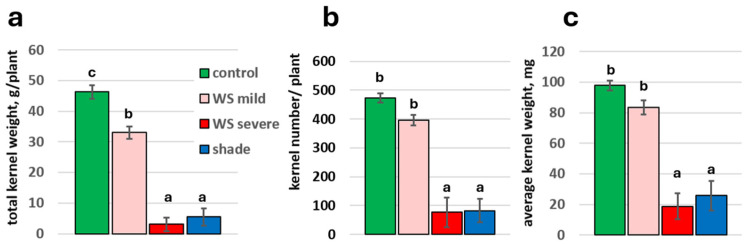
Total kernel dry weight per plant (one ear per plant) (**a**), number of kernels per ear (**b**) and average kernel weight (**c**) in plants subjected to control, WS mild, WS severe, and shade treatments from 0 to 9 DAP. Averages ± SEM are shown; comparisons between treatments that do not have the same letter are significantly (*p* ≤ 0.05) different using the Tukey HSD multiple range test.

After the end of stress treatments, all plants were returned to control conditions and allowed to complete kernel development; they were then harvested at maturity. Mild water stress significantly (*p* ≤ 0.05) decreased total kernel weight per plant at maturity by 30%, whereas severe water stress and shade decreased it by about 90% (Figure 4a). These total weight decreases were due to a combination of decreases in kernel number per plant (Figure 4b) and kernel size (Figure 4c). Given that stress treatments were imposed at early post-pollination, these findings are consistent with the treatments affecting kernel set (versus abortion) and the early development of kernel sink capacity through processes such as endosperm cell division.

### 2.4. Carbohydrate and ABA in Kernel Tissues in Responses to Stress

**Figure 5:**
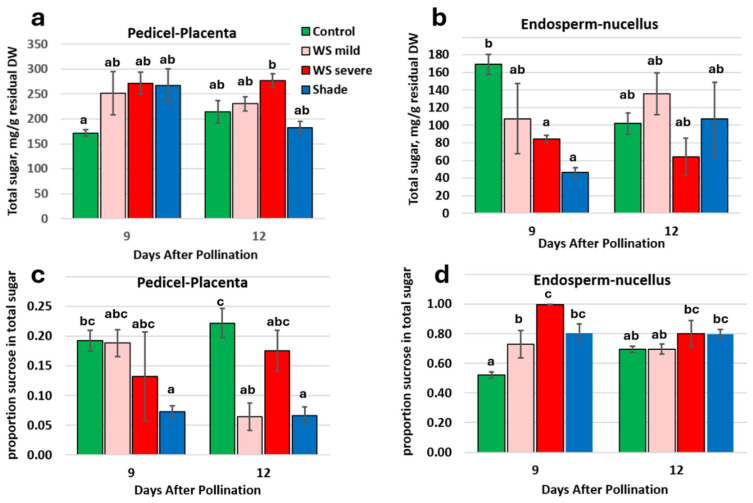
Total sugars in pedicel-placenta (**a**) and endosperm-nucellus (**b**); proportion of sugar as sucrose in pedicel-placenta (**c**) and endosperm-nucellus (**d**) of plants subjected to control, WS mild, WS severe, and shade treatments from 0 to 9 DAP and sampled at 9 and 12 DAP. Averages ± SEM are shown; comparisons between treatments and DAP that do not have the same letter are significantly (*p* ≤ 0.05) different using the Tukey HSD multiple range test.

Stress treatments did not significantly affect sugar content in pedicel-placenta (Figure 5a), but severe WS and shade decreased levels of total sugar in endosperm-nucellus at 9 DAP (Figure 5b). Given that stress slows or halts endosperm growth, the sampled tissues in stress treatments likely contain a high proportion of nucellus while the controls at 9 and 12 DAP largely consist of endosperm. These changes in proportions of tissues represented likely complicate the interpretation of these data. The shade treatment and mild WS significantly (*p* ≤ 0.05) decreased the proportion of total sugar in the form of sucrose in the pedicel-placenta (Figure 5c), whereas the proportion as sucrose in endosperm-nucellus was slightly greater than controls at 9 DAP and was the same in all treatments at 12 DAP (Figure 5d). These changes likely reflect the dynamics of sugar flux relative to metabolism (sucrose hydrolysis and re-synthesis) in these tissues.

**Figure 6:**
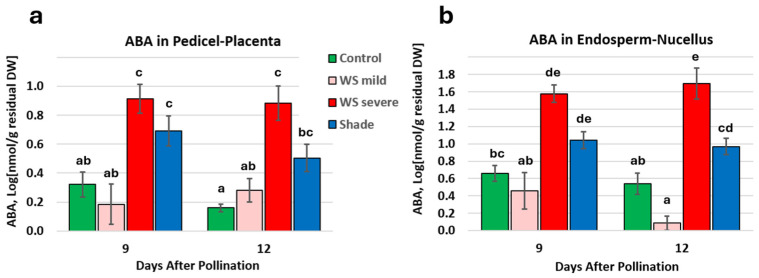
ABA in pedicel-placenta (**a**) and endosperm-nucellus (**b**) of plants subjected to control, WS mild, WS severe, and shade treatments from 0 to 9 DAP and sampled at 9 and 12 DAP. Averages ± SEM are shown; comparisons between treatments and DAP that do not have the same letter are significantly (*p* ≤ 0.05) different using the Tukey HSD multiple range test.

Severe WS increased ABA levels relative to controls in both pedicel-placenta and endosperm-nucellus at 9 and 12 DAP by more than 2.5-fold, as expected for water stressed tissue (Figure 6). However, they were not changed in mild WS. Unexpectedly, shade treatment also increased ABA levels by about 2-fold. This finding indicates that, in these tissues, stimuli other than water potential were able to stimulate ABA accumulation and hence might have triggered ABA responses.

### 2.5. Transcript Expression in Kernel Tissues in Response to Stress

**Figure 7:**
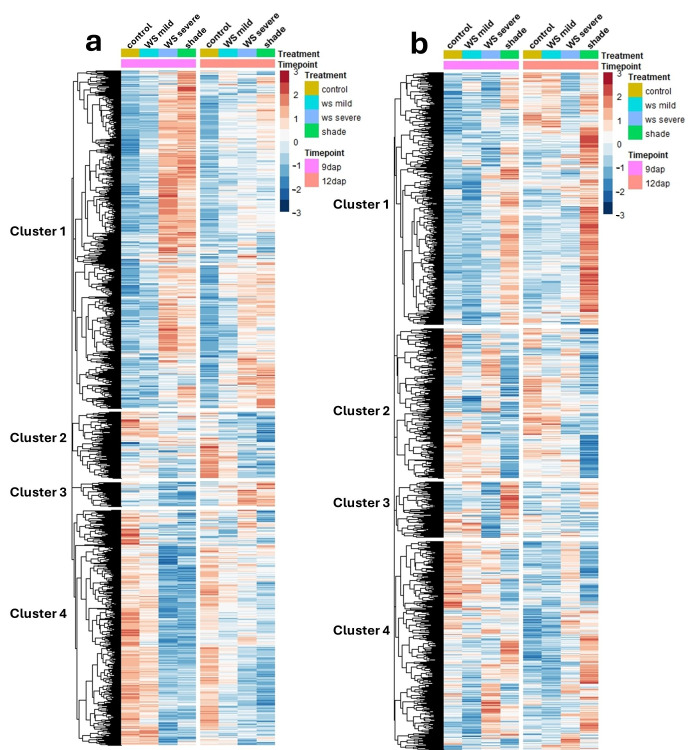
RNA-seq heatmap of gene transcript expression in pedicel-placenta (**a**) and endosperm-nucellus (**b**) of plants subjected to control, WS mild, WS severe, and shade treatments from 0 to 9 DAP and sampled at 9 and 12 DAP. Shown are the expressions for the top 5000 genes, based on variance of WS severe and shade relative to controls at 9 DAP; all of the displayed genes were significantly (Padj ≤ 0.05) different in one or more contrasts between treatments and controls. In each tissue panel, the four columns on the left were sampled at 9 DAP, and the four columns on the right were sampled at 12 DAP. Columns show treatments (left to right): control, WS mild, WS severe, and shade. Colors show the log_2_ of normalized expression. These data, their corresponding gene names, and Padj are shown in Supplementary Table S1.

To provide insight on the underlying physiological bases of stress effects on kernel development, we used RNA-sequencing to profile gene expression in kernel tissues. Differential expression analysis identified a large number of genes in stress treatments whose expression differed significantly (Padj ≤ 0.05: family-wise error rate) from controls. To provide an overall view of these genes, for each tissue, the top 5000 genes based on the magnitude of differential expression at 9 DAP between controls and WS severe or shade are plotted as clustered heatmaps in Figure 7. In pericarp-placenta at 9 DAP, a high proportion of them responded similarly in severe WS and shade (Figure 7a). This was most evident for the stress-upregulated genes in Cluster 1 and the stress-downregulated genes in Cluster 4. Expression in the mild WS treatment was intermediate and often about the same as controls. Expression after recovery at 12 DAP reverted to near control levels for a high proportion of genes, though there were still a large fraction of them with similar expression in severe WS and shade.

In the endosperm-nucellus at both 9 and 12 DAP, there appeared to be a higher number of genes strongly affected by shade than severe WS, especially those upregulated in Clusters 1 and 4 and those downregulated in Clusters 2 and 4 (Figure 7b). In general, mild WS was intermediate between severe WS and control. Genome ontology (GO) enrichment analysis of these clusters indicated that shade-upregulated genes were enriched for endopeptidases and amino acid catabolism, which are associated with senescence, perhaps in aborting kernels, whereas shade-downregulated genes were enriched for starch and carbohydrate metabolism.

### 2.6. Transcript Expression of Carbohydrate Metabolism Genes in Kernel Tissues

**Figure 8:**
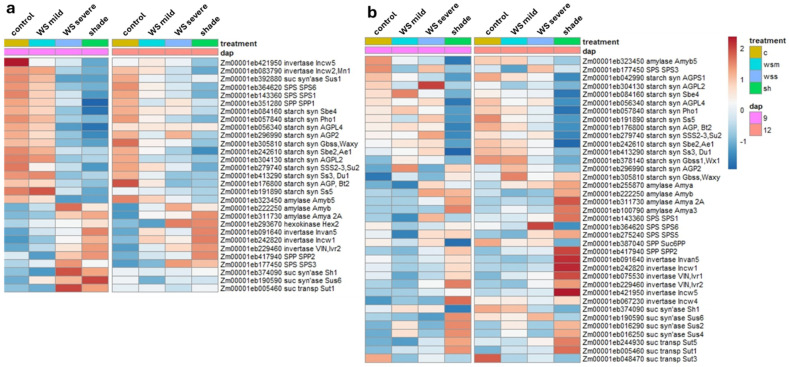
Transcript expression of genes involved in carbohydrate metabolism in pedicel-placenta (**a**) and endosperm-nucellus (**b**) of plants subjected to control, WS mild, WS severe, and shade treatments from 0 to 9 DAP and sampled at 9 (left sub-panels) and 12 (right sub-panels) DAP. Shown are the gene names according to the Zea mays genome assembly B73 RefGen_v5 and a brief description of their functional annotation. All of the displayed genes were significantly (Padj ≤ 0.05) different in one or more contrasts between treatments and controls. Colors show the log_2_ of normalized expression. These data, their corresponding gene names, and Padj are shown in Supplementary Tables S2 and S3.

To provide more focused information for genes in functional categories with more straight-forward interpretation, we analyzed expression of selected genes in specific functional categories. Given that both water stress and shade limit the availability of photosynthate, and the pedicel-placenta is the site of phloem unloading and sugar flux into the growing kernel, we examined expression of genes involved in carbohydrate metabolism (Figure 8). These data indicated that at 9 DAP in the pedicel-placenta a large number of genes encoding enzymes for starch and sucrose synthesis (SPS) were strongly downregulated by both severe WS and shade (Figure 8a). Mild WS was intermediate, though quite similar to controls. Conversely, a group of genes encoding enzymes for starch hydrolysis (amylases) and sucrose hydrolysis (invertases, sucrose synthases) were strongly upregulated by both severe water stress and shade. Although certain genes for sucrose hydrolysis (*Incw5*, *Incw2*, *Sus1*) responded in the opposite direction, and were strongly downregulated, they did so in both severe WS and shade. After relief of stress at 12 DAP, these expression trends subsided and generally returned toward control levels. And expression of these genes in the mild WS treatment was generally intermediate or nearly the same as controls.

In the endosperm-nucellus, expression of genes encoding starch synthesis enzymes and sucrose hydrolysis followed a pattern similar to that in the pedicel-placenta in the shade treatment; however, in the severe WS treatment there was not a clear pattern. The severe WS treatment drastically arrested growth of endosperm-nucellus tissues, whereas the shade had somewhat less effect on turgor mediated expansion growth. This created difficulty in dissecting representative endosperm-nucellus samples in the severe WS and may have compromised the results for this tissue.

### 2.7. Transcript Expression of Trehalose-Related Genes in Kernel Tissues

**Figure 9:**
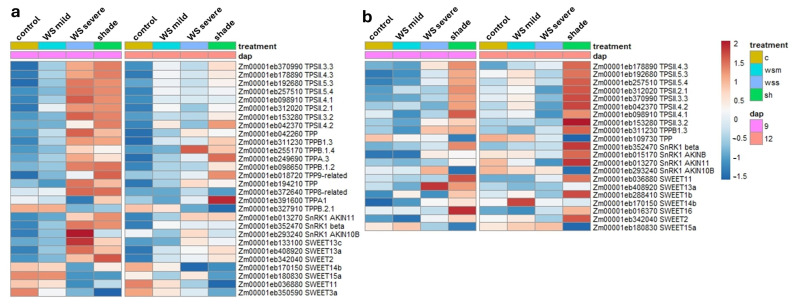
Transcript expression of trehalose-related genes in pedicel-placenta (**a**) and endosperm-nucellus (**b**) of plants subjected to control, WS mild, WS severe, and shade treatments from 0 to 9 DAP and sampled at 9 (left panels) and 12 (right panels) DAP. Shown are the gene names according to the Zea mays genome assembly B73 RefGen_v5 and a brief description of their functional annotation. All of the displayed genes were significantly (Padj ≤ 0.05) different in one or more contrasts between treatments and controls. Colors show the log_2_ of normalized expression. These data, their corresponding gene names and Padj are shown in Supplementary Tables S2 and S3.

Enrichment analysis of differentially expressed genes identified trehalose-related genes as a GO category that were significantly enriched. Indeed, a large family of trehalose metabolism genes were differentially expressed (Figure 9). In pedicel-placenta at 9 DAP, severe WS and shade strongly upregulated expression of eight genes homologous to trehalose-6-phosphate synthase (TPS) while, at the same time, eight genes homologous to trehalose-phosphate phosphatase (TPP) were also upregulated (Figure 9a). Many of these same genes were also upregulated by shade in endosperm. This suggests that the T6P/trehalose pathway was strongly engaged during stress, though the level of T6P would depend on the relative activities of these enzymes. Two homologs of a downstream target of T6P interaction, SnRK1, were also upregulated in pedicel-placenta at 9 DAP. Genes encoding homologs of the sugar transporter SWEET, which is known to be regulated by the T6P/SnRK1 system as well as ABA, had variable responses with some upregulated and others downregulated. Nevertheless, in the pedicel-placenta at 9 DAP, for almost all affected genes the response was nearly always in the same direction for both severe WS and shade. And throughout this panel of genes, mild WS had a slight response relative to controls, intermediate between control and severe WS. The response for the trehalose-related genes in the endosperm was similar to that seen in the pedicel-placenta for the shade treatment, while in the severe WS this was not evident, perhaps due to dissection issues described above.

### 2.8. Transcript Expression of ABA Synthesis and Signaling Genes in Kernel Tissues

**Figure 10:**
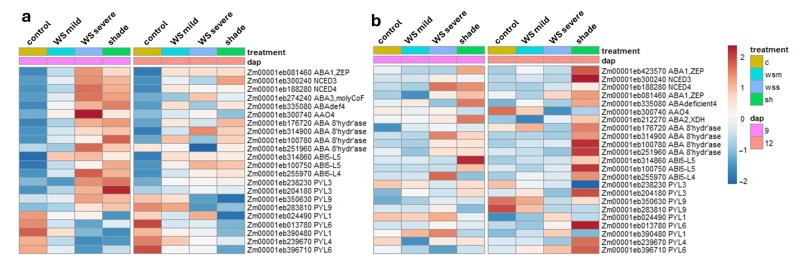
Transcript expression of genes involved in ABA synthesis and signaling in pedicel-placenta (**a**) and endosperm-nucellus (**b**) of plants subjected to control, WS mild, WS severe, and shade treatments from 0 to 9 DAP and sampled at 9 (left panels) and 12 (right panels) DAP. Shown are the gene names according to the Zea mays genome assembly B73 RefGen_v5 and a brief description of their functional annotation. All of the displayed genes were significantly (Padj ≤ 0.05) different in one or more contrasts between treatments and controls. Colors show the log_2_ of normalized expression. These data, their corresponding gene names and Padj are shown in Supplementary Tables S2 and S3.

Several genes involved ABA synthesis and catabolism were upregulated in pedicel-placenta at 9 DAP in both severe WS and shade, with a slight increase in mild WS (Figure 10a). These genes included homologs of enzymes involved in ABA synthesis: the upstream enzymes zeaxanthin epoxidase (*ABA1*), neoxanthin synthase (*ABAdeficient4*), and 9-cis-epoxycarotenoid dioxygenase (NCED), and genes needed for the final step in ABA synthesis, abscisic aldehyde oxidase4 (*AAO4*) and molybdenum cofactor sulfurase (*ABA3*). Also, several homologs of ABA 8′hydroxylase were upregulated by both severe WS and shade. This enzyme is involved in ABA turnover to the inactive catabolite phaseic acid, which serves to fine-tune the ABA levels during an active ABA response [37,38].

In the endosperm-nucellus, some of the ABA metabolism enzymes were upregulated by both severe WS and shade at 9 DAP, but at 12 DAP the expression was strongest in the shade treatment (Figure 10b).

Important ABA signaling genes were also affected by the stress treatments. Homologs of the key transcription factor *ABI5* were upregulated in the pedicel-placenta, and several homologs of the ABA receptor, PYL, were significantly affected by stress treatments. In pedicel-placenta at 9 DAP, homologs of PYL3 and PYL9 were upregulated, whereas PYL1, PYL4 and PYL6 were downregulated in both WS severe and shade. Such differential expression among members of the PYL gene family has been previously observed [39,40], though the present findings emphasize that in the pedicel-placenta both WS severe and shade had parallel responses among this gene family, and the response in mild WS was slight.

### 2.9. Transcript Expression of ABA Responding Genes in Kernel Tissues

**Figure 11:**
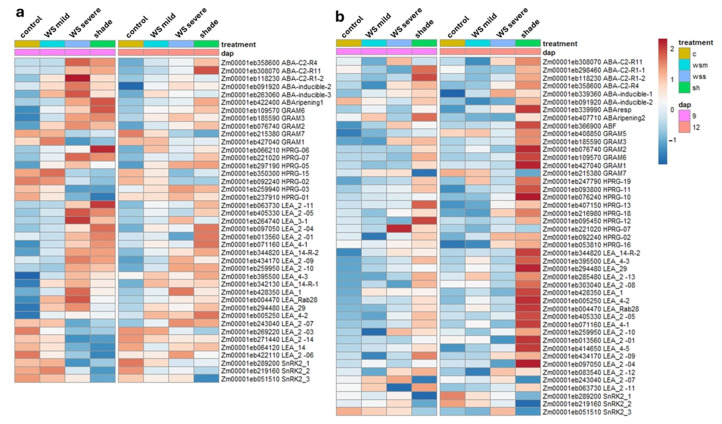
Transcript expression of ABA responding genes in pedicel-placenta (**a**) and endosperm-nucellus (**b**) of plants subjected to control, WS mild, WS severe, and shade treatments from 0 to 9 DAP and sampled at 9 (left panels) and 12 (right panels) DAP. Shown are the gene names according to the Zea mays genome assembly B73 RefGen_v5 and a brief description of their functional annotation. All of the displayed genes were significantly (Padj ≤ 0.05) different in one or more contrasts between treatments and controls. Colors show the log_2_ of normalized expression. These data, their corresponding gene names and Padj are shown in Supplementary Tables S2 and S3.

Among the genes which responded to ABA, homologs in the LEA gene family were well represented (Figure 11). At 9 DAP in the pedicel-placenta, 15 LEA homologs were upregulated while 5 were downregulated by stress (Figure 11a). Notable among the stress-downregulated LEAs is that 4 out of 5 are in the LEA-2 group, which previous work has found to contain several downregulated LEAs [41]. In all cases, LEA homologs responded in the same direction in severe WS and shade, and mild WS was intermediate or slight. Several other homologs of well-known ABA responding genes were also among those upregulated in both severe WS and shade in pedicel-placenta, including genes with the C2 protein domain and GRAM (glucosyltransferases Rab-like GTPase activators) genes. And among genes encoding HRGPs (hydroxyproline-rich glycoproteins), which are structural elements in the cell wall, at 9 DAP in the pedicel-placenta some were upregulated and others were downregulated, with the same direction of response in severe WS and shade in every case.

In the endosperm-nucellus, the response to shade was generally stronger than the response to severe WS, especially at 12 DAP after stress relief (Figure 11b).

### 2.10. Transcript Expression of Ethylene Responding Genes in Kernel Tissues

**Figure 12:**
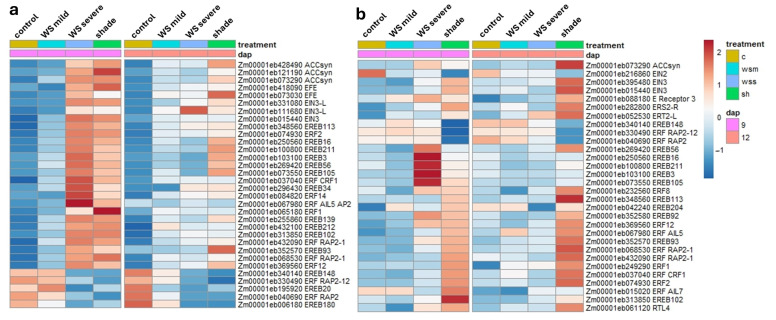
Transcript expression of ethylene response genes in pedicel-placenta (**a**) and endosperm-nucellus (**b**) of plants subjected to control, WS mild, WS severe, and shade treatments from 0 to 9 DAP and sampled at 9 (left panels) and 12 (right panels) DAP. Shown are the gene names according to the Zea mays genome assembly B73 RefGen_v5 and a brief description of their functional annotation. All of the displayed genes were significantly (Padj ≤ 0.05) different in one or more contrasts between treatments and controls. Colors show the log_2_ of normalized expression. These data, their corresponding gene names and Padj are shown in Supplementary Tables S2 and S3.

A large number of ethylene-related genes responded to stress in kernel tissues (Figure 12). In pedicel-placenta tissue at 9 DAP, three homologs of 1-aminocyclopropane-3-carboxylate (ACC) synthase and two homologs of ethylene forming enzyme (EFE, also known as ACC oxidase) were upregulated in both severe WS and shade (Figure 12a). Also, three homologs of *EIN3*, the transcription factor, and master regulator of ethylene signaling were strongly upregulated in response to both of these stress treatments. In addition, expression of a large number of genes encoding proteins in the family of ethylene response transcription factors (ERF/EREB) were significantly affected. Among these ERF genes, stress upregulated 19 of them and downregulated 5 of them; in all cases the direction of response was the same in severe WS and shade, and mild WS was intermediate or slight.

In endosperm-nucellus, shade upregulated an ACC synthase gene and three *EIN3* homologs as well as a large number of ERF homologs (Figure 12b). In endosperm-nucellus at 9 DAP, there was a group of ERFs which were substantially upregulated in severe WS but only slightly in shade (EREB-56, 16, 211,3 and 105), but for the most part shade upregulated more ethylene-related genes than severe WS.

## 3. Discussion

### 3.1. Kernel Set Was Similarly Affected by Water Stress and Shade

The current study subjected maize to water deficit and shade for a brief period, from 1 to 9 days after pollination. Nevertheless, these stress treatments exerted a substantial effect such that kernel set was decreased by more than 80% in the severe water stress and shade treatments (Figure 4). Our goal was to compare the effect of severe WS and shade treatments, and for this purpose the treatments were well matched. These findings are consistent with a large body of evidence for the vulnerability of maize to water deficit at flowering and early post-pollination [2,3]. And studies have shown that shading in field [15,42,43] and greenhouse [29] settings during this stage of development is especially damaging to kernel set and yield. Furthermore, similar responsiveness to water stress and shade has been found in other cereal grains [44,45].

### 3.2. Water Stress and Shade Treatments Differed in Leaf ABA, Sugar Accumulation, and Root Growth

While the severe WS and shade treatments elicited similar effects on kernel development, they differed in several respects: (a) Only the WS treatments accumulated ABA in leaves, with severe WS accumulating much more than mild WS (Figure 1). In contrast, leaves of the shade treatment had baseline levels of ABA, the same as controls. (b) Leaves of shaded plants had low levels of sugars during the treatment, whereas leaf sugars in the mild and severe WS treatments were similar to controls (Figure 2). This tendency to retain or further accumulate sugars in low water potential treatments has been explained as osmotic adjustment to maintain solute levels, retain water, and prevent excess cell shrinkage in the face of low water potentials [46,47]. (c) A third difference between WS and shade responses was in root growth (Figure 3). Given that our experimental setup presented fresh soil in deep soil columns at the time of pollination and the start of treatments, the measured roots represented new root growth after treatment imposition. Severe WS stimulated more root growth and greater root density than well-watered controls, whereas shaded plants had lower root density than controls. Given that shade decreases photosynthesis and availability of assimilates for growth, shade’s effect on root growth is likely due to carbohydrate limitation [48]. For example, recent studies of field-grown maize have shown that 50% shade decreased the number of nodal roots from 5 to 7, which serve the plant at post-pollination stages, and decreased the amount sucrose effluxed by cut roots in a bleeding assay [49]. In contrast, severe WS increased root density relative to controls, even though plants in this treatment are expected to have limited amounts of photoassimilates. This outcome is consistent with a large body of evidence showing that, in response to water deficit, plants prioritize root growth and partition a higher proportion of photoassimilate to root growth [50,51,52]. Studies have indicated that the signaling system that enhances root growth during water deficit may be shoot-derived ABA, which stimulates root growth [53], and compared to shoots, roots have greater sensitivity to ABA during expansion growth such that root growth is favored [51,54]. Our observation that leaf ABA only accumulated in WS treatments (Figure 1) is consistent with the hypothesis that shoot-derived ABA enhanced root growth.

### 3.3. Kernel Tissue Sugar and ABA Levels Respond Similarly to Water Stress and Shade

In contrast to the distinct differences between responses to WS versus shade in leaves and roots, responses to these stresses in kernel tissues were relatively similar. In both the pedicel-placenta and endosperm-nucellus, ABA was significantly (*p* ≤ 0.05) elevated by both severe WS and shade. In the pedicel-placenta, total sugar levels were not significantly (*p* ≤ 0.05) different from controls at 9 and 12 DAP (Figure 5a), while in endosperm-nucellus at 9 DAP, total sugar was less in WS and shade than in controls (Figure 5b). Given that these stresses decrease the supply of photo-assimilate, one might expect that sugar concentration would decrease in kernel tissues. However, while many studies of whole kernels have demonstrated decreases in sugar content in response to WS [12,55] and shade [12], others have indicated that kernel tissues maintain sugar levels in the face of water stress [13,29,31,56] and shade [29]. A possible explanation is that while the kernel tissues as a whole may maintain sugar levels, localized depletion of sugars, such as in the phloem-unloading and transfer pathway in the placenta region, might elicit signaling and a sugar starvation response [23,57].

### 3.4. Transcripts in Pedicel-Placenta Respond Similarly in Water Stress and Shade

To examine responses to WS and shade in the region where phloem terminates near the base of kernels, we dissected out tissue in the pedicel-placenta region and evaluated transcript profiles related to carbohydrate metabolism, transport and signaling. To provide further insight, we also performed transcript profiling on the growing endosperm and surrounding nucellus, though as mentioned above, WS severely diminished kernel size and created difficulties with dissection of this tissue. This limits our ability to make firm conclusions regarding endosperm-nucellus transcript expression. In the pedicel-placenta, consistent with previous reports [11,58], genes encoding enzyme components for starch synthesis were substantially downregulated at 9 DAP in response to WS, whereas genes encoding enzymes for starch hydrolysis and utilization of its carbon were upregulated (Figure 8). Genes in the former category included ADPG-pyrophosphorylases (*AGP*), starch synthase (*Ss*), and starch branching enzyme (*Sbe*), and those in the latter category included α-amylase and β-amylase. Very similar regulation occurred in the photosynthate-limited shade treatment, consistent with previous reports [12].

In addition to enzymes involved in starch hydrolysis, several enzymes involved in sucrose processing and sugar transport were affected by the stress treatments. Shen et al. [55] used in-depth data mining of spatio-temporal transcriptomes in maize kernels to identify transporter genes specifically expressed in the placenta tissues where phloem unloading and carbohydrate transfer to developing endosperm take place. Given the complexity of the localized steps in sugar metabolism and transport, there are numerous genes and sometimes contrary regulation. Consistent with previous studies [59,60,61], WS and shade downregulated cell wall invertases *INCW2* and *INCW5* in the pedicel-placenta, whereas vacuolar invertase *Ivr2* was upregulated (Figure 8a). In addition, WS and shade increased expression of sugar membrane transporters *Sut1* and several SWEETs, as others have reported [55]. Ruan [62] suggested that the cell wall invertases and sugar transporters, which are often specifically expressed in placenta and basal endosperm apoplasmic interfaces, may be involved in both providing carbon nutrients as well as sugar signals. Indeed, studies of genetic silencing of cell wall invertases in Arabidopsis (*Arabidopsis thaliana* (L.) Heynh) suggest their primary function might be in developing sugar signals to regulate ovule development [63].

Considerable evidence has indicated that trehalose metabolism is a pivotal signaling system in kernel tissues [15,31,49,58,64,65,66,67]. Consistent with this, we observed substantial upregulation of several genes in pedicel-placenta tissues encoding trehalose phosphate synthase (TPS) and trehalose phosphate phosphatase (TPP) in both WS and shade treatments (Figure 9). Furthermore, three SnRK1 targets of T6P signaling were upregulated by stress, consistent with previous reports for maize placental tissues [68,69]. While the immediate effect of sugar starvation is expected to be activation of pre-existing SnRK1 when T6P levels drop, prolonged sugar starvation has been observed to upregulate SnRK1 and its downstream targets, such as SWEETs and *Sut1* [68].

In addition to its role in the sugar starvation response, studies have indicated that the trehalose regulatory system interacts with stress ABA. In wheat (*Triticum aestivum*, L), a QTL affecting kernel size was identified as a gene encoding a TPP, and when it was overexpressed, ABA synthesis (NCED) and ABA receptor (PYL) genes were upregulated [70]. This response provides a possible explanation for our observation that shade increased the levels of ABA in kernel tissues (Figure 6), even though the treatment had no effect on ABA synthesis in leaves (Figure 1). Indeed, in the pedicel-placenta, both WS and shade upregulated NCED, three other ABA synthesis pathway genes, and four PYL genes encoding ABA receptor/signaling proteins (Figure 11). Furthermore, in pedicel-placenta, a host of ABA responding genes, including 14 LEA genes and several GRAM and HPRG genes, were upregulated in both WS and shade. GRAM proteins (glucosyltransferases, Rab GTPase-activating proteins) and HPRG proteins (hydroxyproline-rich glycoproteins) have promoters that are highly enriched with cis-acting ABRE (ABA-Responsive Elements) and are therefore indicators of ABA upregulation.

Several genes related to ethylene responses were also upregulated in both WS and shade (Figure 12). These findings further extend observations of maize kernels by Shen et al. [11], where ethylene emission increased 2- to 5-fold from 4 to 12 DAP in water deficit treatments. Our findings also provide further gene expression support for findings by Shen et al. [12], where WS and shade similarly increased ethylene emission and decreased kernel set, whereas altering the sugar status with partial ear pollination decreased competition for carbohydrate and partially alleviated stress effects. Among the upregulated genes in our study were several involved in synthesis of ethylene and initial signaling. In pedicel-placenta, we found that three ACC synthase genes and two homologs of ethylene forming enzyme (EFE) were upregulated by severe WS and shade in the pedicel-placenta. Also, three *EIN3* (a transcription factor involved in the ethylene response pathway) and 19 ERF and EREB proteins (ethylene response factor and ethylene response element binding factor) were upregulated similarly in pedicel-placenta at 9 DAP by both WS and shade. A large family of ERFs are known [71], some which are upregulated and others downregulated during ethylene responses, in accordance with tissue and developmental-stage specificity [72]. Indeed, we found that five ERF/EREB genes were downregulated, and remarkably, both WS and shade affected them similarly. Shen et al. [11,12] noted that ethylene has previously been implicated in grain abortion in cereals [73,74,75], raising the possibility that ethylene works with other signaling cross-talk to abort carbohydrate-starving kernels. Indeed, Shi et al. [35] provide evidence that ARGOS proteins interact with the ethylene receptor signaling-complex; ARGOS variants, created by CRISPR-Cas9, improve maize grain yield under field drought conditions [36,76].

While the transcript-level findings in the present investigation provide valuable insight into regulation in response to WS and shade, additional regulation at the post-transcriptional, translational, and post-translational steps are likely. These possibilities merit future investigation.

The insights gained from this work provide an improved understanding of the fundamental processes important for adapting agricultural practices and breeding strategies to a changing climate. Because the optimization of planting density and canopy architecture affects photosynthate supply to sink organs, and these factors interact with water status, the responses elucidated in this study are highly relevant to crop management. As recent reviews have emphasized, breeding for future climates requires accounting for multiple interacting factors [5,6,8,9]. By clarifying underlying physiological processes, this research offers a framework to guide future crop improvement efforts.

## 4. Conclusions

**Figure 13:**
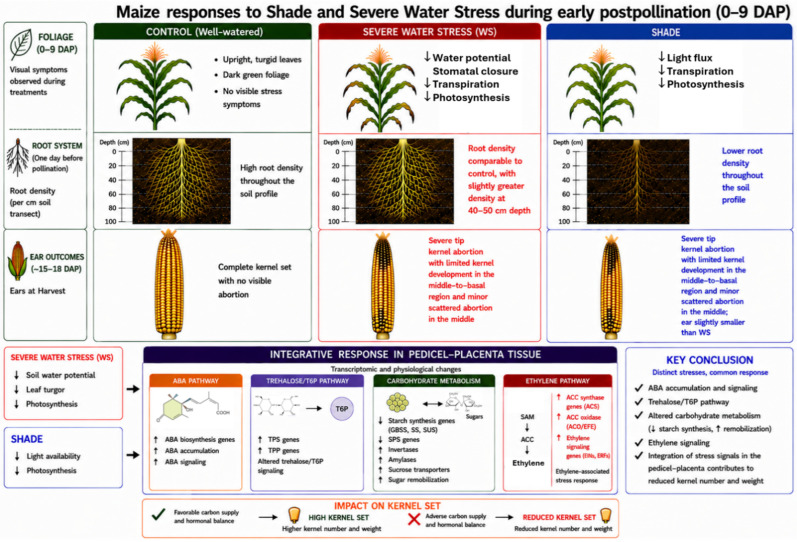
Summary of the effects of severe water stress and shade observed in this study.

Post-pollination water stress and shade treatments, which had similar effects on kernel yield per plant, had a remarkable similarity in the profile of genes which were significantly different from controls. Overall, a high proportion of the top 5000 affected genes were similarly upregulated or downregulated in the pedicel-placenta of severe WS and shade treatments. This provides strong evidence that there is a common underlying basis for their effects on kernel set. In contrast, other responses were specific to each stress: leaf ABA was only elevated in response to WS, leaf sugar levels were only depleted in shade treatment, and root density decreased in shaded plants whereas it increased in severe WS plants. Our studies indicate that responses in pedicel-placenta may play key roles in kernel set vs. abortion decisions, while expression in endosperm-nucellus may reflect growth and development that were difficult to sort out without a more micro-anatomical experimental approach. The expression of genes in the pedicel-placenta in key functional categories suggested a possible model of mechanistic signaling which leads to kernel abortion. A hypothesis consistent with the data is that photosynthate flux into the kernel decreases during stress, which may be detected in the phloem unloading zone in the pedicel-placenta and lead to changes in trehalose metabolism and signal low carbohydrate status. This may trigger numerous changes, including increased starch breakdown, arrested starch synthesis, and altered sucrose and hexose metabolism and transport. Furthermore, while it is well established that low water potential stimulates ABA synthesis under water stressed (WS) conditions, the present data suggest that in the pedicel-placenta, low carbohydrate availability, either directly or via interactions with the ethylene signaling pathway, may also elevate ABA synthesis in plants of both WS and shade treatments. These mechanisms remain hypotheses that warrant further empirical verification. The combined cross-talk of these coordinated transcriptional changes are associated with stress-induced kernel abortion as summarized in Figure 13. The current work contributes to our understanding of the postulated common underlying basis of multiple stresses by which limited photosynthate availability leads to reproductive failure and loss of crop yield. Improved understanding of this system may provide insight into possible targets for future crop improvement.

## 5. Materials and Methods

### 5.1. Plant Material

Seed of the single-cross commercial maize hybrid HL 2020, which matures in short seasons [75 d relative maturity, 945 heat units to physiological maturity (10 °C base)], was obtained from Hyland Seeds (Blenheim, ON, Canada).

### 5.2. Growth Conditions

Plants were germinated and initially grown two plants per pot in 7 L starter pots (Poly-Tainer #2), containing soil-less rooting media consisting of 63% (*v*/*v*) sphagnum peat moss, 25% (*v*/*v*) vermiculite, and 12% (*v*/*v*) perlite (amorphous alumina silicate, Whittemore Co., Lawrence, MA, USA), to which 2.8 g/L (*w*/*v*) of microfine dolomitic limestone (National Lime and Stone Co., Findlay OH, USA), 2.2 g/L (*w*/*v*) of 10-5-10 Jacks Pro Media fertilizer mix plus III (J.R. Peters, Inc., Allentown, PA, USA), and 2.2 g/L of powdered CaSO_4_ were added. The plants were grown in glasshouse conditions at thermostatic set-point day/night temperatures of 30/25 °C and supplemental lighting from 400 W high pressure sodium lamps (Plantmax Grow Lamp MPN: PX-LU400, 1000Bulbs Co., Garland, TX, USA), spaced on a 1.8 by 1.0 m grid which was automatically engaged between 600 and 1800 when solar photosynthetic (400–700 nm) photon flux density was <500 μmol m^−2^ s^−1^. After 4 weeks of growth, soluble NPK fertilizer was added weekly with irrigation water (15-5-15 Cal-Mg Special with 80% of N as NO_3_, J.R.Peters, Inc.). When husks of primary ears emerged, they were bagged to control pollination, and 2–4 d after silk emergence silks were pollinated with abundant pollen. One day before pollination, plants and root systems were removed intact from starter pots and transplanted to bottomless pots which, in turn, were firmly placed onto a bed of rooting media in deep pots. Deep pots were constructed with black high-density polyethylene 1.0 m tall and 19.5 cm in diameter (31 L), which had smooth interior surfaces and exterior ribs for mechanical reinforcement, similar to those previously described [50]. To enable intermittent viewing and photography of roots, each pot had a 15 cm panel (circumferential width) cut-out from top to bottom, which was fitted with 1.5 mm thick transparent PVC, and in turn fitted with a black exterior panel to exclude light. Deep pots were filled with rooting media which was precisely prepared with 1.2 kg H_2_O/kg of dry media, thereby creating a rooting-media water potential of −0.18 MPa, as measured by calibrated psychrometry [50].

### 5.3. Treatments

Treatments were randomly assigned within each batch; control and shade had 6 replicate batches; mild and severe water deficit had three replications. Pots were sealed around stems to prevent direct evaporation from the soil surface. The weight of plants + pots was determined daily and used to calculate evapotranspiration. Plants in the control and shade treatments were rewatered daily and weighed again. At one d after pollination, plants for the shade treatment were placed under a coarse-weave black polypropylene shade-cloth which was suspended vertically to form a 1.5 m dia., 4.8 m height, open-top cylindrical enclosure which excluded 70% of solar radiation. Plants in the water stress treatments were given limited water each day so that soil water was gradually depleted. Depletion of soil water in the mild water stress treatment began at one d after pollination (DAP) and soil water was allowed to decline 0.3 kg H_2_O/d such that pot weight was 2.5 kg less than well-watered pots by 9 DAP. The severe water stress treatment was initiated one d before pollination, and by 3 DAP, pot weight was 2 kg less than well-watered pots; by 9 DAP pot weight had declined 2.8 kg. At 9 DAP, shaded plants were removed from shade, and water stress plants were rewatered and watered normally until maturity.

### 5.4. Data/Sample Collection

Leaf samples: Leaf tissue samples were collected daily using a sharpened 6 mm diameter hole puncher. Two leaf disks were taken from upper leaves per plant and were immediately placed in tubes with 0.3 mL of ice-chilled 80% (*v*/*v*) ethanol–water solution at 0 °C. Samples were initially stored at −18 °C, then dried at 40 °C.

Transpiration rates: Each treatment plant + pot was weighed daily at 900 to 1100; plants were irrigated as described above under Treatments and weighed again. Evapo-transpiration rates were determined by calculating daily weight differences.

Endosperm and placenta-pedicel sampling: At 9 and 12 DAP, kernel samples were collected for RNA sequencing and metabolite analysis. At each sampling, husks were peeled back on one quarter of the circumference of the ear to expose kernels in the apical end of the ear. The pericarp was removed from the kernel apex by cutting transversely along 2–3 kernel rows with a razor blade. At 9 DAP, the transverse cut removed the apical one half to 2/3 of the kernel so that the nucellus was removed from the apical portion of the kernel, leaving the endosperm, embryo and traces of nucellus; at 12 DAP, the transverse cut removed about 1/5th of the apical portion of the kernel, again leaving most of the endosperm and embryo. Hereinafter, these samples will be called endosperm since embryos had little development at this stage and represented a small fraction of the total. The sampled tissue also included some of the surrounding nucellus. Endosperm samples were collected with a scooping motion using a narrow spatula and immediately transferred into porous polyester bags (tea bag) suspended in liquid nitrogen. Once endosperm samples were obtained, the kernels were cut transversely once more to a depth immediately above the basal endosperm bowl. Any excess endosperm was first carefully scooped off and discarded, and then placenta-pedicel samples were collected with a cut at the pedicel and placed into tea bags suspended in liquid nitrogen. Tea bags were then transferred into a larger dewar of liquid nitrogen to maintain cold storage before being finally stored in a −80 °C freezer.

Root Data Collection: Using the clear viewing panels, root photos were taken at 9 and 12 days after pollination. The root zone was shaded to reduce glare, and all photos included a 100 cm measuring stick positioned from the soil surface to allow pixel scaling and depth measurements during later analysis. Image J software version 1.54j, (https://imagej.net/ij/; accessed 1 May 2024) was used to determine angle with respect to horizontal and root density across linear zones at each depth as follows: images were uploaded into the software, pixel scale was transformed to centimeters using the included measuring stick in each image, and panel widths were determined to ensure equivalent viewings across treatments. Root density and angle was measured at 30, 40, 50, 60, and 70 cm depths for images taken at 9 DAP. In each photo, at each depth, a straight line was drawn horizontally across the viewed panel and all roots which intersected the line were counted. Total counts were divided by panel width to determine root counts per cm, or root linear density. Root angle was determined by using the Image J straight line tool. Lines were traced over each root to indicate their angles with respect to horizontal lines, and at the location where roots intersected with horizontal depth lines, their angles, between 0° and 90°, were measured.

### 5.5. Carbohydrate Analysis

Leaf and placenta-pedicel material was extracted twice with 80% (*v*/*v*) ethanol–water; combined extracts were transferred to 96-well plates and dried at 40 °C. Remaining insoluble tissue debris was treated with amyloglucosidase (E.C. 3.2.1.3) and α-amylase at 40 °C for 24 h to hydrolyze starch to glucose. Glucose in extracts and in starch hydrolysates was assayed as described by Ober et al. [30] by coupled enzyme assay with glucose oxidase + peroxidase and para-hydroxybenzoic acid + 4-aminoantipyrene, which reacts in linear proportion to glucose with absorbance measured at 490 nm. Sucrose was assayed as glucose released following hydrolysis by invertase. Glucose and sucrose standards were used to calibrate the assays.

### 5.6. ABA Assay

After carbohydrate analysis, the remaining portion of leaf and placenta-pedicel extracts were transferred into 96-well plates and dried at 40 °C. Samples were redissolved in 30% methanol–water and fractionated with reverse-phase quick-chromatography in columns packed with 25 mg of 40 µm dia C18-silica material (Supelco Discovery DSC-18 SPE; Sigma-Aldrich, St. Louis, MO, USA) as described by Setter et al. [28]. Samples were analyzed with an enzyme-linked immunosorbent assay (ELISA) [28]. Briefly, in this method the ABA concentration of a sample was determined by an inverse relationship between the amount of anti-ABA monoclonal antibody (mouse monoclonal antibody, PDM 09347, Agdia, Elkhart, IN, USA) bound to free ABA analyte in a sample versus ABA conjugate immobilized to each well of 96-well polystyrene plates (Corning High-Binding 9018 plates, Corning, Inc., Tewksbury, MA, USA). After washing the unbound antibody and ABA from the wells, the amount of bound monoclonal antibody was measured using a secondary antibody made in goat that targets the mouse antibody and to which alkaline phosphatase was conjugated. The bound alkaline phosphatase was quantified by its activity in hydrolyzing para-nitrophenol phosphate whose product was measured by its absorbance at 405 nm. The assay was calibrated with (+)ABA standards. Each sample had two technical replicates, and their averages were used for statistical analysis of the biological replicates.

### 5.7. Statistical Analysis

Mixed-model ANOVA was used, which included the fixed effects of treatment, sampling date, and sampling date by treatment interaction. Batches (repetitions at different times), which accounted for batch-to-batch variation, were modeled as random effects. The lm and ANOVA functions of the “stats” package conducted in R studio (v2023.12.0; R version 4.0.3) were used to model the effects [77]. The “emmeans” package (v1.7.5) [78] was used for mean comparisons with multiple tests using Tukey–Kramer honest significant difference tests.

### 5.8. RNA Extraction

Total RNA was extracted from endosperm and placenta-pedicel samples using a procedure that included CTAB detergent and purified on silica RNA columns as previously described [79]. Samples were ground in a mortar and pestle chilled with liquid N_2_; about 0.15 g of the powder was vortexed vigorously for 5 min with 1 mL of extraction buffer containing 1% [*w*/*v*] CTAB detergent, 100 mM Tris-HCl [pH 8.0], 1.4 M NaCl, 20 mM EDTA, and 2% [*v*/*v*] 2-mercaptoethanol. The suspension was mixed for 1 min with 0.2 mL of chloroform to remove detergent, lipophilic constituents, and debris; the tubes were centrifuged and the top layer containing RNA was moved to a new tube, and 0.7 mL of a buffer containing 4 M guanidine thiocyanate, 10 mM MOPS (pH 6.7) and 0.5 mL of 100% ethanol (100%) was added and mixed. This mixture was applied to silica RNA columns (RNA mini spin column, Epoch Life Science, Missouri City, TX, USA) to which RNA was bound. Using vacuum, fractions were eluted sequentially to remove undesired solutes with (a) 0.75 mL of buffer (10 mM MOPS-HCl [pH 6.7] with 1 mM EDTA, containing 80% [*v*/*v*] ethanol–water); (b) 0.75 mL of 80% ethanol–water (twice); and (c) with centrifugation at 10,000 *g*, RNA was eluted with 20 µL of RNAase-free water. The RNA quality was evaluated with a gel system (TapeStation 2200, Agilent Technologies, Santa Clara, CA, USA).

### 5.9. 3′RNA Sequencing and Quantitative Expression Analysis

The 3′RNA-seq libraries were prepared at the Cornell Genomics facility (https://www.biotech.cornell.edu/core-facilities-brc/facilities/genomics-facility, accessed on 9 July 2024) from about 500 ng total RNA per sample using the Lexogen QuantSeq 3′ mRNA-Seq Library Prep Kit FWD for Illumina (Greenland, NH, USA; https://www.lexogen.com/quantseq-family/, accessed on 9 July 2024). The libraries were randomly pooled for maximum evenness and sequenced on one lane of an Illumina NextSeq500 sequencer, single-end 1×86bp, and de-multiplexed based upon six base i7 indices using Illumina bcl2fastq2 software (version 2.18; Illumina, Inc., San Diego, CA, USA). Samples with fewer than 200,000 demultiplexed reads and genes with an average count of less than two across all samples were excluded from further analysis. For the remaining samples, Illumina adapters on the 3′ end and the first twelve bases on the 5′ end (corresponding to the random primer) were removed from the de-multiplexed fastq files using Trimmomatic (version 0.36) [80]. Poly-A tails and poly-G stretches of ≥10 bases in length were then removed using the BBDuk program from the package BBMap (https://sourceforge.net/projects/bbmap/; version 37.50, accessed on 9 July 2024), keeping reads at least 18 bases in length after trimming. The trimmed reads were aligned to the Zea mays genome assembly B73 RefGen_v5 (https://download.maizegdb.org/Zm-B73-REFERENCE-NAM-5.0/Zm-B73-REFERENCE-NAM-5.0.fa.gz, accessed on 9 September 2024) using the STAR aligner (version 2.7.0f) [81], allowing a read to map in at most 10 locations with at most 6% mismatches, while filtering out all non-canonical intron motifs. The number of RNAseq reads for each sample and the percentage of reads mapped to the reference genome are shown in Supplement Table S4. The output SAM files were converted to BAM using SAMtools (version 1.15.1), and the number of reads overlapping each gene in the gff3 file on the forward strand were counted using HTSeq-count (version 0.6.1).

### 5.10. Differential Expression Analysis

Analysis of differential gene expression was accomplished using the DESeq2 package, version 1.36.0 [82], which adjusts *p*-values in multiple testing (Padj) due to the large number of tests. Data were modeled with DAP and treatment as fixed effects and batch as a random effect. Contrasts were performed between each treatment (WS mild, WS severe, and shade) versus control at each sampling date (9 DAP and 12 DAP), and genes with Padj ≤ 0.05 were identified. Shrinkage was by apeglm, and further processing and generation of heatmaps was done with Bioconductor software, version 3.22 in R [83]. To obtain overall lists of top genes in each tissue type, genes which were significantly (Padj ≤ 0.05) different between WS severe or shade and control at either 9 or 12 DAP were identified, and then, to obtain a list that encompassed the genes with strong statistical support and variation among samples, these genes were further filtered to obtain the top 5000 genes after scaling them by variance from controls. Lists of genes representing various functional categories were obtained via searches of functional annotation in Phytozome 14 (https://phytozome-next.jgi.doe.gov/; accessed on 3 February 2026).

## Data Availability

The original contributions presented in this study are included in the article/Supplementary Material. Further inquiries can be directed to the corresponding author.

## Supplementary Materials

The following supporting information can be downloaded at: https://www.mdpi.com/article/10.3390/plants15142130/s1, Table S1: Top 5000 differentially expressed genes; Table S2: Differential expression contrasts and statistics; Table S3: Differentially expressed genes in select categories, carbohydrate metabolism, trehalose, ABA synthesis and signaling, ABA responding, and ethylene response; Table S4: RNA sample information.

## Abbreviations

The following abbreviations are used in this manuscript:

DAP	Days after pollination
WS	Water stress
ABA	Abscisic acid
SEM	Standard error of the mean

## References

[1] Claassen M.M., Shaw R.H. (1970). Water Deficit Effects on Corn. II. Grain Components. Agron. J..

[2] Campos H., Cooper M., Edmeades G.O., Loffler C., Schussler J.R., Ibanez M. (2006). Changes in drought tolerance in maize associated with fifty years of breeding for yield in the US corn belt. Maydica.

[3] Grant R.F., Jackson B.S., Kiniry J.R., Arkin G.F. (1989). Water Deficit Timing Effects on Yield Components in Maize. Agron. J..

[4] Barker T., Campos H., Cooper M., Dolan D., Edmeades G., Habben J., Schussler J., Wright D., Zinselmeier C., Janick J. (2005). Improving Drought Tolerance in Maize. Plant Breeding Reviews.

[5] Simmons C.R., Lafitte H.R., Reimann K.S., Brugière N., Roesler K., Albertsen M.C., Greene T.W., Habben J.E. (2021). Successes and insights of an industry biotech program to enhance maize agronomic traits. Plant Sci..

[6] Messina C., Ciampitti I.A., Berning D., Bubeck D., Hammer G., Cooper M. (2022). Sustained improvement in tolerance to water deficit accompanies maize yield increase in temperate environments. Crop Sci..

[7] Messina C., McDonald D., Poffenbarger H., Clark R., Salinas A., Fang Y., Gho C., Tang T., Graham G., Hammer G.L. (2021). Reproductive resilience but not root architecture underpins yield improvement under drought in maize. J. Exp. Bot..

[8] Prasanna B.M., Burgueño J., Beyene Y., Makumbi D., Asea G., Woyengo V., Tarekegne A., Magorokosho C., Wegary D., Ndhlela T. (2022). Genetic trends in CIMMYT’s tropical maize breeding pipelines. Sci. Rep..

[9] He Z., Zhang P., Jia H., Zhang S., Nishawy E., Sun X., Dai M. (2024). Regulatory mechanisms and breeding strategies for crop drought resistance. New Crops.

[10] Echarte L., Tollenaar M. (2006). Kernel set in maize hybrids and their inbred lines exposed to stress. Crop Sci..

[11] Shen S., Liang X.-G., Zhang L., Zhao X., Liu Y.-P., Lin S., Gao Z., Wang P., Wang Z.-M., Zhou S.-L. (2020). Intervening in sibling competition for assimilates by controlled pollination prevents seed abortion under postpollination drought in maize. Plant Cell Environ..

[12] Shen S., Li B.-B., Deng T., Xiao Z.-D., Chen X.-M., Hu H., Zhang B.-C., Wu G., Li F., Zhao X. (2020). The equilibrium between sugars and ethylene is involved in shading- and drought-induced kernel abortion in maize. Plant Growth Regul..

[13] Schussler J.R., Westgate M.E. (1995). Assimilate flux determine kernel set at low water potential in maize. Crop Sci..

[14] Schussler J.R., Westgate M.E. (1991). Maize kernel set at low water potential II. Sensitivity to reduced assimilates at pollination. Crop Sci..

[15] Liang X.-G., Gao Z., Shen S., Paul M.J., Zhang L., Zhao X., Lin S., Wu G., Chen X.-M., Zhou S.-L. (2020). Differential ear growth of two maize varieties to shading in the field environment: Effects on whole plant carbon allocation and sugar starvation response. J. Plant Physiol..

[16] Setter T.L., Flannigan B.A. (1989). Relationship between Photosynthate Supply and Endosperm Development in Maize. Ann. Bot..

[17] Pagano E., Cela S., Maddonni G.A., Otegui M.E. (2007). Intra-specific competition in maize: Ear development, flowering dynamics and kernel set of early-established plant hierarchies. Field Crops Res..

[18] Paponov I.A., Sambo P., Erley G.S.a.m., Presterl T., Geiger H.H., Engels C. (2005). Grain yield and kernel weight of two maize genotypes differing in nitrogen use efficiency at various levels of nitrogen and carbohydrate availability during flowering and grain filling. Plant Soil.

[19] DeBruin J.L., Schussler J.R., Mo H., Cooper M. (2017). Grain Yield and Nitrogen Accumulation in Maize Hybrids Released during 1934 to 2013 in the US Midwest. Crop Sci..

[20] DeBruin J.L., Hemphill B., Schussler J.R. (2018). Silk Development and Kernel Set in Maize as Related to Nitrogen Stress. Crop Sci..

[21] Vasmatkar P., Kaur K., Pannu P.P.S. (2021). Field based assessment of yield-related traits and flowering response in Zea mays towards Southern corn leaf blight. Indian Phytopathol..

[22] Battaglia M.L., Lee C., Thomason W. (2018). Corn Yield Components and Yield Responses to Defoliation at Different Row Widths. Agron. J..

[23] McLaughlin J.-E., Boyer J.-S. (2004). Glucose localization in maize ovaries when kernel number decreases at low water potential and sucrose is fed to the stems. Ann. Bot..

[24] Zinselmeier C., Lauer M.J., Boyer J.S. (1995). Reversing drought-induced losses in grain yield: Sucrose maintains embryo growth in maize. Crop Sci..

[25] Boyle M.G., Boyer J.S., Morgan P.W. (1991). Stem infusion of liquid culture medium prevents reproductive failure of maize at low water potential. Crop Sci..

[26] Zhang L., Li X.-H., Gao Z., Shen S., Liang X.-G., Zhao X., Lin S., Zhou S.-L. (2017). Regulation of maize kernel weight and carbohydrate metabolism by abscisic acid applied at the early and middle post-pollination stages in vitro. J. Plant Physiol..

[27] Yu L.-X., Setter T.L. (2016). Comparative transcriptomes between viviparous1 and wildtype maize developing endosperms in response to water deficit. Environ. Exp. Bot..

[28] Setter T.L., Yan J., Warburton M., Ribaut J.-M., Xu Y., Sawkins M., Buckler E.S., Zhang Z., Gore M.A. (2011). Genetic association mapping identifies single nucleotide polymorphisms in genes that affect abscisic acid levels in maize floral tissues during drought. J. Exp. Bot..

[29] Setter T.L., Flannigan B.A., Melkonian J. (2001). Loss of kernel set due to water deficit and shade in maize: Carbohydrate supplies, abscisic acid, and cytokinins. Crop Sci..

[30] Ober E.S., Setter T.L., Madison J.T., Thompson J.F., Shapiro P.S. (1991). Influence of Water Deficit on Maize Endosperm Development 1: Enzyme Activities and RNA Transcripts of Starch and Zein Synthesis, Abscisic Acid, and Cell Division. Plant Physiol..

[31] Oury V., Caldeira C.F., Prodhomme D., Pichon J.-P., Gibon Y., Tardieu F., Turc O. (2016). Is Change in Ovary Carbon Status a Cause or a Consequence of Maize Ovary Abortion in Water Deficit during Flowering?. Plant Physiol..

[32] Oury V., Tardieu F., Turc O. (2016). Ovary Apical Abortion under Water Deficit Is Caused by Changes in Sequential Development of Ovaries and in Silk Growth Rate in Maize. Plant Physiol..

[33] An J., Almasaud R.A., Bouzayen M., Zouine M., Chervin C. (2020). Auxin and ethylene regulation of fruit set. Plant Sci..

[34] Müller M., Munné-Bosch S. (2015). Ethylene Response Factors: A Key Regulatory Hub in Hormone and Stress Signaling. Plant Physiol..

[35] Shi J., Drummond B.J., Wang H., Archibald R.L., Habben J.E. (2016). Maize and Arabidopsis ARGOS Proteins Interact with Ethylene Receptor Signaling Complex, Supporting a Regulatory Role for ARGOS in Ethylene Signal Transduction. Plant Physiol..

[36] Shi J., Habben J.E., Archibald R.L., Drummond B.J., Chamberlin M.A., Williams R.W., Lafitte H.R., Weers B.P. (2015). Overexpression of ARGOS Genes Modifies Plant Sensitivity to Ethylene, Leading to Improved Drought Tolerance in Both Arabidopsis and Maize. Plant Physiol..

[37] Ji X., Dong B., Shiran B., Talbot M.J., Edlington J.E., Hughes T., White R.G., Gubler F., Dolferus R. (2011). Control of abscisic acid catabolism and abscisic acid homeostasis is important for reproductive stage stress tolerance in cereals. Plant Physiol..

[38] Vallabhaneni R., Wurtzel E.T. (2010). From epoxycarotenoids to ABA: The role of ABA 8′-hydroxylases in drought-stressed maize roots. Arch. Biochem. Biophys..

[39] Fan W., Zhao M., Li S., Bai X., Li J., Meng H., Mu Z. (2016). Contrasting transcriptional responses of PYR1/PYL/RCAR ABA receptors to ABA or dehydration stress between maize seedling leaves and roots. BMC Plant Biol..

[40] He Z., Zhong J., Sun X., Wang B., Terzaghi W., Dai M. (2018). The Maize ABA Receptors ZmPYL8, 9, and 12 Facilitate Plant Drought Resistance. Front. Plant Sci..

[41] Zaidi I., Soltani N., Saidi M.N. (2026). Genome-Wide Analysis of LEA Genes Family in Barley with Emphasis on their Role as Molecular Chaperones Under Abiotic Stress. J. Plant Growth Regul..

[42] Setter T.L., Flannigan B.A. (1986). Sugar and Starch Redistribution in Maize in Response to Shade and Ear Temperature Treatment1. Crop Sci..

[43] Espelet F., Rotili D.H., D’Andrea K.E., Maddonni G.A. (2025). Critical periods for the expression of vegetative and reproductive plasticity in maize crops. Field Crops Res..

[44] Okawa S., Makino A., Mae T. (2003). Effect of irradiance on the partitioning of assimilated carbon during the early phase of grain filling in rice. Ann. Bot..

[45] Rajala A., Hakala K., Mäkelä P., Muurinen S., Peltonen-Sainio P. (2009). Spring wheat response to timing of water deficit through sink and grain filling capacity. Field Crops Res..

[46] Turner N.C. (2018). Turgor maintenance by osmotic adjustment: 40 years of progress. J. Exp. Bot..

[47] Blum A. (2017). Osmotic adjustment is a prime drought stress adaptive engine in support of plant production. Plant Cell Environ..

[48] Devaux C., Baldet P., Joubes J., Dieuaide Noubhani M., Just D., Chevalier C., Raymond P. (2003). Physiological, biochemical and molecular analysis of sugar-starvation responses in tomato roots. J. Exp. Bot..

[49] Liu J.D.M., Liu S., Ma Y., Qin Z., Liu C., Wang R. (2025). Anatomical and Physiological Responses of Maize Nodal Roots to Shading Stress and Nitrogen Supply. Agronomy.

[50] Duque L.O., Setter T.L. (2013). Cassava Response to Water deficit in Deep Pots: Root and Shoot Growth, ABA, and Carbohydrate Reserves in Stems, Leaves and Storage Roots. Trop. Plant Biol..

[51] Sharp R.-E., Poroyko V., Hejlek L.-G., Spollen W.-G., Springer G.-K., Bohnert H.-J., Nguyen H.-T. (2004). Root growth maintenance during water deficits: Physiology to functional genomics. J. Exp. Bot..

[52] Rostamza M., Richards R.A., Watt M. (2013). Response of millet and sorghum to a varying water supply around the primary and nodal roots. Ann. Bot..

[53] McAdam S.A.M., Brodribb T.J., Ross J.J.C.P.C.E.R. (2016). Shoot-derived abscisic acid promotes root growth. Plant Cell Environ..

[54] Saab I.N., Sharp R.E., Pritchard J. (1992). Effect of inhibition of abscisic-acid accumulation on the spatial-distribution of elongation in the primary root and mesocotyl of maize at low water potentials. Plant Physiol..

[55] Shen S., Ma S., Chen X.-M., Yi F., Li B.-B., Liang X.-G., Liao S.-J., Gao L.-H., Zhou S.-L., Ruan Y.-L. (2022). A transcriptional landscape underlying sugar import for grain set in maize. Plant J..

[56] Setter T.L., Parra R. (2010). Relationship of carbohydrate and abscisic acid levels to kernel set in maize under postpollination water deficit. Crop Sci..

[57] Makela P., McLaughlin J.E., Boyer J.S. (2005). Imaging and quantifying carbohydrate transport to the developing ovaries of maize. Ann. Bot..

[58] Paul M.J., Gonzalez-Uriarte A., Griffiths C.A., Hassani-Pak K. (2018). The Role of Trehalose 6-Phosphate in Crop Yield and Resilience. Plant Physiol..

[59] Andersen M.N., Asch F., Wu Y., Jensen C.R., Næsted H., Mogensen V.O., Koch K.E. (2002). Soluble Invertase Expression Is an Early Target of Drought Stress during the Critical, Abortion-Sensitive Phase of Young Ovary Development in Maize. Plant Physiol..

[60] Jiang Z., Piao L., Guo D., Zhu H., Wang S., Zhu H., Yang Z., Tao Y., Li M., Liu C. (2021). Regulation of Maize Kernel Carbohydrate Metabolism by Abscisic Acid Applied at the Grain-Filling Stage at Low Soil Water Potential. Sustainability.

[61] Zinselmeier C., Sun Y., Helentjaris T., Beatty M., Yang S., Smith H., Habben J. (2002). The use of gene expression profiling to dissect the stress sensitivity of reproductive development in maize. Field Crops Res..

[62] Ruan Y.-L. (2022). CWIN-sugar transporter nexus is a key component for reproductive success. J. Plant Physiol..

[63] Liao S., Wang L., Li J., Ruan Y.-L. (2020). Cell Wall Invertase Is Essential for Ovule Development through Sugar Signaling Rather Than Provision of Carbon Nutrients1 [OPEN]. Plant Physiol..

[64] Nuccio M.L., Wu J., Mowers R., Zhou H.-P., Meghji M., Primavesi L.F., Paul M.J., Chen X., Gao Y., Haque E. (2015). Expression of trehalose-6-phosphate phosphatase in maize ears improves yield in well-watered and drought conditions. Nat. Biotechnol..

[65] Bledsoe S.W., Henry C., Griffiths C.A., Paul M.J., Feil R., Lunn J.E., Stitt M., Lagrimini L.M. (2017). The role of Tre6P and SnRK1 in maize early kernel development and events leading to stress-induced kernel abortion. BMC Plant Biol..

[66] Acosta-Pérez P., Camacho-Zamora B.D., Espinoza-Sánchez E.A., Gutiérrez-Soto G., Zavala-García F., Abraham-Juárez M.J., Sinagawa-García S.R. (2020). Characterization of Trehalose-6-phosphate Synthase and Trehalose-6-phosphate Phosphatase Genes and Analysis of its Differential Expression in Maize (Zea mays) Seedlings under Drought Stress. Plants.

[67] Oszvald M., Primavesi L.F., Griffiths C.A., Cohn J., Basu S.S., Nuccio M.L., Paul M.J. (2018). Trehalose 6-Phosphate Regulates Photosynthesis and Assimilate Partitioning in Reproductive Tissue. Plant Physiol..

[68] Akbudak M.A., Yildiz K., Cetin D., Filiz E., Yukselbaba U., Srivastava V. (2025). Characterization of ZmSnRK1 genes and their response to aphid feeding, drought and cold stress. Genet. Resour. Crop Evol..

[69] Kakumanu A., Ambavaram M.M.R., Klumas C., Krishnan A., Batlang U., Myers E., Grene R., Pereira A. (2012). Effects of Drought on Gene Expression in Maize Reproductive and Leaf Meristem Tissue Revealed by RNA-Seq. Plant Physiol..

[70] Liu H., Si X., Wang Z., Cao L., Gao L., Zhou X., Wang W., Wang K., Jiao C., Zhuang L. (2023). TaTPP-7A positively feedback regulates grain filling and wheat grain yield through T6P-SnRK1 signalling pathway and sugar–ABA interaction. Plant Biotechnol. J..

[71] Qi H., Liang K., Ke Y., Wang J., Yang P., Yu F., Qiu F. (2023). Advances of Apetala2/Ethylene Response Factors in Regulating Development and Stress Response in Maize. Int. J. Mol. Sci..

[72] Cheng C., An L., Li F., Ahmad W., Aslam M., Ul Haq M.Z., Yan Y., Ahmad R.M. (2023). Wide-Range Portrayal of AP2/ERF Transcription Factor Family in Maize (Zea mays L.) Development and Stress Responses. Genes.

[73] Hays D.-B., Do J.-H., Mason R.E., Morgan G., Finlayson S.-A. (2007). Heat stress induced ethylene production in developing wheat grains induces kernel abortion and increased maturation in a susceptible cultivar. Plant Sci..

[74] Yang J., Zhang J., Liu K., Wang Z., Liu L. (2006). Abscisic acid and ethylene interact in wheat grains in response to soil drying during grain filling. New Phytol..

[75] Yang J., Zhang J., Wang Z., Liu K., Wang P. (2006). Post-anthesis development of inferior and superior spikelets in rice in relation to abscisic acid and ethylene. J. Exp. Bot..

[76] Shi J., Gao H., Wang H., Lafitte H.R., Archibald R.L., Yang M., Hakimi S.M., Mo H., Habben J.E. (2016). ARGOS8 variants generated by CRISPR-Cas9 improve maize grain yield under field drought stress conditions. Plant Biotechnol. J..

[77] R_Core_Team (2017). R: A Language and Environment for Statistical Computing. https://www.R-project.org/.

[78] Lenth R. (2019). Emmeans: Estimated Marginal Means, Aka Least-Squares Means.

[79] Hyde P.T., Setter T.L. (2022). Long-day photoperiod and cool temperature induce flowering in cassava: Expression of signaling genes. Front. Plant Sci..

[80] Bolger A.M., Lohse M., Usadel B. (2014). Trimmomatic: A flexible trimmer for Illumina sequence data. Bioinformatics.

[81] Dobin A., Davis C.A., Schlesinger F., Drenkow J., Zaleski C., Jha S., Batut P., Chaisson M., Gingeras T.R. (2012). STAR: Ultrafast universal RNA-seq aligner. Bioinformatics.

[82] Love M.I., Huber W., Anders S. (2014). Moderated estimation of fold change and dispersion for RNA-seq data with DESeq2. Genome Biol..

[83] Love M., Anders S., Kim V., Huber W. (2015). RNA-Seq workflow: Gene-level exploratory analysis and differential expression. F1000Research.

